# All about the RNA after all

**DOI:** 10.7554/eLife.24106

**Published:** 2017-01-24

**Authors:** Tatjana Trcek, Ruth Lehmann

**Affiliations:** 1 Skirball Institute of Biomolecular Medicine, Department of Cell Biology, Howard Hughes Medical Institute, NYU School of Medicine, New York, United States; 1 Skirball Institute of Biomolecular Medicine, Department of Cell Biology, Howard Hughes Medical Institute, NYU School of Medicine, New York, United Stateslehmann@saturn.med.nyu.edu

**Keywords:** RNA granules, P granules, phase separation, MEX-5, MEG-3, germline, germ granules, germ cells, *C. elegans*

## Abstract

RNA molecules cause the proteins involved in the formation of germ granules to coalesce into liquid droplets.

**Related research article** Smith J, Calidas D, Schmidt H, Lu T, Rasoloson D, Seydoux G. 2016. Spatial patterning of P granules by RNA-induced phase separation of the intrinsically-disordered protein MEG-3. *eLife* **5**:e21337. doi: 10.7554/eLife.21337

The embryo of any organism that reproduces sexually must develop germ cells, such as those that go on to become egg and sperm cells in animals. This is because these are the only cells that are destined to transfer genetic material to the next generation. One characteristic of developing germ cells is the presence of particles termed “germ granules” (Voronina et al., 2011). Made from various RNA and protein molecules, these granules are believed to regulate the translation of messenger RNA (mRNA) molecules inside the germ cells during development (Seydoux and Braun, 2006).

Many of the components that are found in germ granules are conserved between distantly related species. Studies in this area have commonly involved the roundworm *Caenorhabditis elegans*, which, like other animals, starts life as a single fertilized egg or zygote. At first, germ granules are spread uniformly throughout this cell. However, as the zygote starts to develop a distinct front and back, the germ granules are only found in the back of the zygote: this is why the germ granules in *C. elegans* are called P granules (with “P” being short for the P lineage of cells that forms at the posterior). This process is repeated during further cell divisions, such that the P granules continue to segregate into those cells that will eventually give rise to the germ cells. Now, in eLife, Geraldine Seydoux and colleagues at the Johns Hopkins University School of Medicine – including Jarrett Smith as first author – report how two RNA-binding proteins with opposing effects control where P granules form (Smith et al., 2016).

Early explanations as to why P granules segregated asymmetrically were based on the idea that they were actively transported to the posterior half. However, a few years ago, it was noted that proteins found in germ granules could spontaneously de-mix from the cytoplasm and coalesce to form germ granules (Brangwynne et al., 2009). This phenomenon, called a phase transition, resembles how oil droplets form when oil is mixed with water. However, only the granules that formed in the posterior of the zygote were stable in *C. elegans*, and any granules that started to form in the front half disappeared instead.

P granules only grow in the posterior, in part, because a gradient of RNA-binding proteins somehow restricts where they can form (Griffin et al., 2011; Schubert et al., 2000). This raises some questions: how is a protein gradient transformed into an on-off switch for P granule formation? And what triggers the phase transition so that P granules are only stable in the posterior?

Some proteins in germ granules contain “intrinsically disordered regions” that lack a well-defined three-dimensional structure (Kato et al., 2012; Courchaine et al., 2016; Hyman et al., 2014). Smith et al. now demonstrate that two intrinsically disordered, RNA-binding proteins – namely MEG-3 and its homolog MEG-4 – lie at the heart of P granule formation, and that MEG-3 is essential for germ granules to nucleate. In vitro, MEG-3 will spontaneously assemble into aggregates, but only at concentrations higher than those found in the zygote (Figure 1). However, Smith et al. discovered that this phase transition was enhanced when RNA is present. As such, simply varying the RNA levels in a test tube or in the zygote can change when and where P granules form. Smith et al. also showed that another RNA-binding protein called MEX-5 (which is not a component of P granules) competes with MEG-3 for access to the RNA, and that the high concentrations of MEX-5 at the front end of the zygote prevent P granules being formed there (Figure 1).

**Figure 1.**
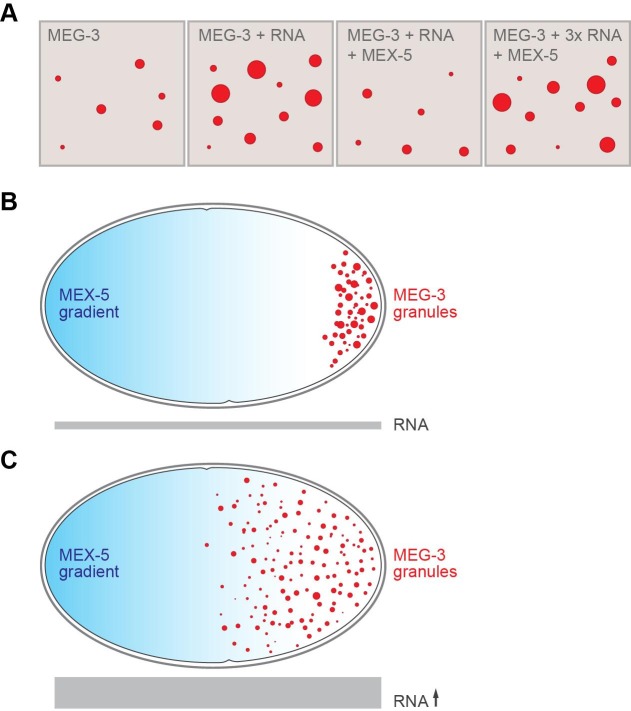
RNA and the formation of P granules. (**A**) The formation of liquid droplets of the protein MEG-3 (red circles) in vitro is enhanced by RNA (second and fourth panels) and antagonized by the protein MEX-5 (third panel). (**B**) In the single-celled zygote, the front of the cell (left) has higher levels of MEX-5 (blue shading) than the rear of the cell (right). MEX-5 and MEG-3 both bind to RNA, and competition between them restricts the formation of P granules to the regions where the concentration of MEX-5 is low (that is, to the posterior end of the cell). (**C**) If the RNA levels in the cell (represented by the area of the gray bar) are increased (by blocking an RNA degradation pathway), more P granules are formed, and they also form further forward in the zygote than normal.

FIGURE CREDIT: Alexey Soshnev, Tatjana Trcek and Ruth Lehmann.

A recent theoretical study in *C. elegans* proposed a similar mechanism, with MEX-5 and a P granule protein called PGL-3 competing to bind to mRNA molecules (Saha et al., 2016). However, Smith et al. show that PGL-3 is not essential for the nucleation of P granules, and that it is not needed to establish the asymmetric distribution of granules either. It is therefore more likely that MEG-3 forms a critical scaffold for the P granule and then recruits other P granule proteins, including PGL-3 (Hanazawa et al., 2011; Wang et al., 2014).

MEX-5 and MEG-3 bind to RNA with little specificity (Pagano et al., 2007; Smith et al., 2016), but the adaptor proteins found in germ cells might make it possible for these proteins to bind to different sets of mRNAs (Weidmann et al., 2016). This selective binding could establish a gradient of specific mRNAs that runs from the front to the back of the zygote, with critical mRNAs being captured at the end of the cell that goes on to become the germ cells (Gallo et al., 2010; Lehmann, 2016; Seydoux and Braun, 2006).

RNA-protein granules are widespread in nature. They are, in fact, found in every cell in the human body, and likely regulate RNAs in many different ways (Couchrane, et al., 2016). Phase transitions might drive the formation of these other granules too, similar to P granule formation in *C. elegans*. These granules often contain RNA-binding proteins with intrinsically disordered regions and are also enriched with RNAs (Han et al., 2012; Lin et al., 2015; Schwartz et al., 2013; Teixeira et al., 2005; Zhang et al., 2015). As such, many of them may likewise rely on RNAs to form. The new mechanism reported by Smith et al. could explain how a variety of RNA-protein granules end up sorted into different areas of the cell, even though they share multiple components.
